# Health inequalities among homeless adolescents*

**DOI:** 10.1590/1518-8345.6250.3756

**Published:** 2022-11-07

**Authors:** Edna Johana Mondragón-Sánchez, Patricia Neyva da Costa Pinheiro, Lorena Pinheiro Barbosa

**Affiliations:** 1 Universidade do Quindio, Faculdade de Ciências da Saúde, Armênia, Quindio, Colombia.; 2 Universidade Federal do Ceará, Faculdade de Enfermagem, Fortaleza, Ceará, Brazil.

**Keywords:** Nursing, Adolescent, Vulnerable Populations, Homeless People, Social Determinants of Health, Contextual Effects of Health Disparities

## Abstract

**Objective:**

highlight health inequalities of homeless adolescents based on the conceptual framework of social determinants of health.

**Method:**

this is a convergent parallel mixed-methods study. The population consisted of adolescents who are homeless, with purposeful sampling and data saturation. Quantitative data were obtained with a questionnaire and qualitative data through semi-structured interviews.

**Results:**

19 homeless adolescents participated in the study, 13 (68.4%) aged 16 to 19 years; 11 (57.9%) were male, 3 (15.8%) were female, and 5 (26.3%) were transgender adolescents. Participants experienced different levels of exposure and vulnerabilities to conditions that affect health and were directly related to intermediate determinants of health.

**Conclusion:**

this study provides an understanding of the inequalities related to the health of homeless adolescents and shows evidence that supports strategies to promote equity and dignity in health care.

## Introduction

Adolescents who are homeless represent a clear and extreme evidence of social inequality. It is a problem of multiple dimensions, not only due to its causes, but also due to the population heterogeneity. Many adolescents who are homeless are marked by deep ruptures with their families and their educational, professional and social environment, which, combined with the conditions of poverty, make them experience precarious realities in society^1^.

Homeless adolescents consider the streets their space of life and are faced with different situations of social and economic exclusion, deprived of human dignity and well-being. This reality has been present for at least one century in Latin America^2^, considering streets mean for adolescents a space of freedom, where everything is allowed. However, it involves the cruel aspect of denial of rights - such as education, health, leisure - and family and community life. Adolescents who are homeless show the failure of society to protect these people from situations of vulnerabilities and risks^3^.

The concept of inequality is described in texts of international organizations related to human rights and, more specifically, in studies about rights of adolescents such as: the right to freedom, respect and dignity; right to family and community life; right to practice a profession and protection at work; education, culture, sport and leisure; and, finally, protection from physical or psychological violence^4^.

In general terms, the rights of adolescents are described in the United Nations Convention on the Rights of the Child, which has its moral foundation in the fulfillment of universal needs such as: physical nutrition, autonomy, celebration, integrity, interdependence, play, and spiritual communion. Then, the convention aims to ensure the absence of inequalities^4^ ^-^ ^5^.

Most diseases and resulting inequalities are related to the conditions in which people are born, live, grow, work, and age. This group of factors is called social determinants of health (SDH), a term that covers economic, political, cultural, and environmental dimensions^6^ ^-^ ^8^.

Thus, the conceptual framework of the World Health Organization (WHO) for social determinants of health can be used to explore and identify how the social, economic, and political context of a country affects the socioeconomic positions in society, where population is stratified by social class, gender and sexual identity, ethnicity, income, education, and occupation^8^ ^-^ ^10^.

Even so, an individual’s socioeconomic position shapes specific determinants of health status, known as intermediate determinants of health, which include material and psychosocial circumstances, such as housing, availability of food, living conditions, social support, and biological and behavioral factors^6^ ^-^ ^8^.

In general, health professionals have difficulties handling people in situations of vulnerability. In this sense, it is important to produce studies on this topic so that nursing has a strategic role in health care in this context. In the literature, this topic has not been fully explored, and such evidence would be relevant to qualify care and then enhance social integration of homeless adolescents, as this is a risk group in the current context^11^.

Nursing is a critical profession for health promotion and education, as it constitutes a strategic field of action to improve health conditions and the risk factors of adolescents who are homeless, which influence their health and are a result of the SDH. Public social policy restructuring is essential to ensure all important resources to members of society to compensate for income inequalities^5^.

Then, the health needs of adolescents can be fulfilled with holistic care, covering body, mind, emotion, spirit, and environment. Therefore, a more personal relationship with the human being is established, under the care of the nurse, increasing trust and making the person more receptive to health guidance, which offers the possibility for patients to be responsible for their own health and, then, improve their quality of life. This study provides an opportunity to review the conditions of other people, such as homeless adolescents, considering their SDH, enabling changes in their lives focused on health promotion.

Therefore, this study helped recognize adolescents as actors and social subjects, with possibilities to improve their ability to reflect on the context by encouraging their development and adoption of healthy behaviors^5^ ^,^ ^9^ ^-^ ^10^. Regarding knowledge gaps, there is a lack of nursing studies assessing the health needs of homeless adolescents, the interpersonal relationships between homeless adolescents and the professionals who provide care, and their personal experiences in the context of vulnerability and risk^12^. Considering the above, this article aims to highlight health inequalities of homeless adolescents based on the conceptual framework of social determinants of health.

## Method

### Study design

This is a convergent parallel mixed-methods study^13^ ^-^ ^16^.

### Study period and site

This study was conducted from June 2020 to June 2021, with the application of interviews at bus terminals, supermarkets, malls, streets, squares, beaches, busy avenues, commercial and tourist areas. This project was articulated with social educators from the *Ponte de Encontro* Program, which approaches adolescents on the street and is part of the *Fundação da Criança e da Família Cidadã* (Funci), linked with the city administration of Fortaleza, Ceará.

### Population and sample

The population consisted of homeless adolescents, with purposeful sampling and using datasaturation as the criterion for the number of observations. The sample consisted of a heterogeneous group in terms of gender identity, age, race, and life stories, with a context in common: extreme poverty, broken or weakened family bonds^3^ ^-^ ^17^.

### Data collection procedures and instruments

**Figure 1:**
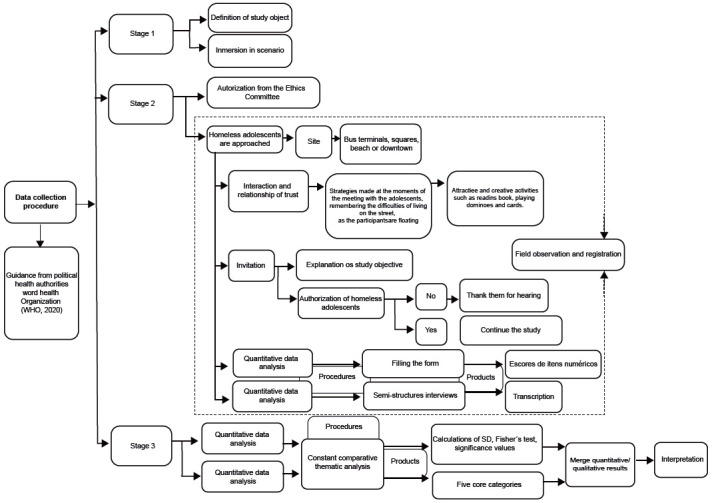
Data collection procedures

Source: Developed according to the Guidelines for research with homeless children and adolescents (2018) and Creswell and Clark^14^

Data collection was performed in three stages: definition of the object, field work, and data analysis (Figure 1). The procedures are described below.

Quantitative data were obtained using a questionnaire with sociodemographic questions; and qualitative data were obtained in a semi-structured interview.

### Data analyses

The analysis of quantitative data was conducted with the Statistical Package for the Social Sciences (SPSS) software, version 25.0 for Windows. Inferential tests were applied using Fisher’s test and the chi-square test. The analysis of qualitative data was performed using a discourse analysis supported by reflections on the frameworks of social determinants of health, with the help of the ATLAS.ti9 software, License nº. L-24E-AD0. For qualitative data, a systematic coding was adopted, as described below^18^.

Open coding: analytical procedure through which data were segmented and opened to show the thoughts, ideas, and meanings of the adolescents in order to discover, label, and develop concepts. The inductive steps were conducted and data categories were used without previous conditions.

Axial coding: it was produced by establishing hierarchical relationships with the subcategory of properties and dimensions around the category considered as the axis, thus obtaining a scheme to help understand the phenomena and configure the core categories.

Selective coding: it enabled the conceptual and theoretical relationship between codes and subcategories, that is, the theory. The interviewees are represented by the letter “E” and numbered successively as they were included in the study in order to preserve their identities.

### Ethical aspects

This study was approved by the Research Ethics Committee (REC) of Universidade Federal do Ceará, under approval nº 4.430.75/2020, and meets the ethical and legal requirements of Resolution 466/12 and Resolution 510 of the National Health Council^19^ ^-^ ^20^. All adolescents were properly informed about the study objectives. Participants were asked to read and sign the consent form; however, as many of them could not read, the researcher read it to them and recorded their oral acceptance. For adolescents with parents and/or legal guardians, they also signed the informed consent form.

## Results

Nineteen homeless adolescents participated in the study; 13 (68.4%) were aged 16 to 19 years. Regarding gender identity, 11 (57.9%) were male, 3 (15.8%) were female, and 5 (26.3%) were transgender adolescents. Regarding their skin color, 15 (78.9%) self-reported as non-white (brown and black). About their origin, 14 (73.7%) were from Fortaleza. About how long they have been homeless, 9 (47.4%) reported 2 to 12 years and 10 (52.6%), six months. No participant had been homeless between 6 and 12 months. For weekly income, 10 (52.6%) adolescents received BRL 100 to 180 and 9 (47.4%), BRL 200 to 300.

Homeless adolescents experienced different levels of exposure and vulnerabilities to conditions that affect health and were directly related to intermediate determinants of health. According to data of our study, the core category of *health inequality among homeless adolescents* had two subcategories: *health system* and *health and well-being*.

### Health system

The health system is seen as the organized social response to the health issues of a given population, providing access to health care services to people with illnesses and health problems. Although the right to health is recognized as a human right, our study, it is not exercised in an equitable and full manner by all population in Brazil. According to the participants, this perception is associated with the type of care provided, the characteristics of illnesses and health problems, the places individuals go to in case of health problems, and the lack of and ID document (Table 1).

Regarding the possession of an ID document, only 9 (47.4%) adolescents had a birth/marriage certificate and 5 (26.3%) had the ID card and a birth/marriage certificate. The lack of an ID document influences the provision of health care, because, as illustrated in the statements below, care was refused to the adolescents. One of them left the health center and his health condition became worse on the streets:

*[…]* No, but it seems that I have mental problems. We don’t have a problem with living on the streets, we just have these silly things of people in our own house fighting, but apart from that, we sleep well *[...]*. Most of the time I stay on the streets, because I need a document and I don’t have it *(E19)*.

Regarding the possession of an ID document and its association with age group, gender identity and race, no statistically significant association was observed between these variables (Table 2).

**Table 1:** Sociodemographic data: illnesses and health problems, health care centers, and ID document. Fortaleza, CE, Brazil, 2021 (n=19)

Characteristic	Frequency	Percentage	Cumulative percentage
ID document possession			
Birth/marriage certificate	9	47.4	47.4
ID card	2	10.5	57.9
ID card and birth/marriage certificate	5	26.3	84.2
ID card, CPF^*^	2	10.5	94.7
No document possession	1	5.3	100.0
Illnesses and health problems of Homeless adolescents			
Yes	9	47.4	47.4
No	10	52.6	100.0
Place they go when they have health problems			
Public health center	8	42.1	42.1
Hospital/UPA^†^	9	47.4	89.5
Does not know/remember	2	10.5	100.0

*CPF = Brazilian taxpayer ID; ^†^UPA = Emergency care unit

**Table 2:** Bivariate analysis of the association between ID document possession, age group, gender identity, and race. Fortaleza, CE, Brazil, 2021 (n=19)

Characteristic	Test	Value	Degrees of freedom	P-value
ID document/Age group	Pearson’s chi-square	1.310	1	0.252
	Likelihood ratio	1.326	1	0.250
	Linear-by-linear association	1.241	1	0.265
	Number of valid cases	19		
ID document/Race	Pearson’s chi-square	6.632	4	0.157
	Likelihood ratio	7.250	4	0.123
	Linear-by-linear association	0.010	1	0.920
	Number of valid cases	19		
Illnesses and health problems on the streets/Age group	Pearson’s chi-square	3.161	4	0.531
	Likelihood ratio	4.603	4	0.330
	Linear-by-linear association	0.934	1	0.334
	Number of valid cases			
Illnesses and health problems on the streets/Gender identity	Pearson’s chi-square	4.852	2	0.088
	Likelihood ratio	6.125	2	0.047
	Linear-by-linear association	4.546	1	0.033
	Number of valid cases	19		
Illnesses and health problems on the street/Race	Pearson’s chi-square	1.552	1	0.213
	Likelihood ratio	1.598	1	0.206
	Linear-by-linear association	1.470	1	0.225
	Number of valid cases	19		

Regarding illnesses and health problems on the streets, our study showed that 10 (52.6%) adolescents reported no health problems, while 9 (47.4%) reported some illness or condition (Table 1). According to the following statements, the most frequent illnesses and health problems were: malaria, mycosis, gastritis, respiratory problems, type 1 diabetes, and mental illnesses such as depression: 

[…] *No, I just have gastritis and breathing problems* (E9).[…] *No, I just have psychological problems* […]*. Yeah, I was diagnosed with depression, anxiety crisis, and panic disorder* […]*. Yeah, when I have a crisis, I go there right away so I can get better.* […] *Only medical follow-up, because I have type 1 diabetes* […]. *Exactly, then my leg hurts because I had an allergy to the sun and it ended up hurting a lot and, sometimes, I eat unhealthy food and it takes time to heal* (E18). […] *It’s mycosis* (E19).

The homeless population has increased in recent years in Brazil^21^; so, understanding their perception of health care is the first step to meet the needs in a humanized way and create a bond between this population and the health service, considering that several diseases can be aggravated by the fact that they live on the streets.

Regarding the place they go to when they feel sick, nine (47.4%) adolescents go to the hospital/emergency care unit (UPA) and 8 (42.1%) prefer to go to a health care center - basic health unit (Table 1). Their statements mention these both places, and some choose not to go anywhere:

*[…]* When I get sick, I don’t tell anyone, there is prejudice at the UPA *[…]*. I just lie down *(E4)*. *[…]* Sometimes I go to a health care center to get some medication, but sometimes they don’t have it *[…]*. Yes, health care center *(E6)*.

Their preference for the health care center is evident in the statements, as they report worse care provided at UPA:

*[...]* When I have even a toothache, I go [to the health care center]. They treat me well, I get there, they ask for the document, then they write down my name, I don’t know what *[...]*. Only at the UPA, I found it bad, because at UPA, I got there and waited for two, three hours and they couldn’t see me and I left, I think they don’t like us, people who live on the streets *[...]*. I think health care centers are better, so when I have a simple problem, I go to there, they resolve my problem, quickly, and it’s quick *(E16)*.

Health and well-being

Well-being refers to attitudes and/or behaviors that improve the quality of life to reach an optimal health state. Then, the complexity of health and well-being is undeniable, regardless of the perspective from which these aspects are approached, especially when considering homeless adolescents. The fragility of health and well-being is associated with the conditions of the places where people sleep, who they sleep with, quality/quantity of sleep, rent (if applicable), street food, and personal hygiene (Table 3).

**Table 3:** Characteristics of the places where adolescents sleep, who they sleep with, quality/quantity of sleep, rent, street food, and personal hygiene. Fortaleza, CE, Brazil, 2021 (n=19)

Characteristic	Frequency	Percentage	Cumulative percentage
Sleep at a house	6	31.6	31.6
Sleep on the street	13	68.4	100.0
Sleep alone	11	57.9	57.9
Sleep with a partner	8	42.1	100.0
Buy own food	12	63.2	63.2
Eat at an accessible restaurant	7	36.8	100.0
Personal hygiene on the street	4	21.1	21.1
Personal hygiene in public toilets	11	57.9	78.9
Personal hygiene at parents’ house/their house	4	21.1	100.0

Our study found that 13 (68.4%) adolescents sleep on the street and have several places to sleep, such as the beach, under viaducts, on squares, and at bus terminals. Six teenagers sleep at home like to be on the streets during the day to make money during this period. Regarding who they sleep with, 11 (57.9%) adolescents sleep alone and 8 (42.1%) sleep with a partner (Table 2). The statements below reinforce the permanent inequalities experienced by adolescents:

*[…]* I used to sleep under one viaduct, I looked for a safe area *(E1)*. *[…]* I slept under a boat, it was turned upside down, then I slept under it, alone *(E3)*. *[…]* Some days I sleep on Praça do Ferreira with my partner *[…]*. Near the beach, in Dragão do Mar, a place where we are safe *(E6)*.

Regarding the sleep quality/quantity, the interviewees report that it is not good, as the street context has violence, unquietness, and unexpected and risky situations, which can occur while they are sleeping. Therefore, they feel fear when they sleep at night, so they prefer to sleep during the day:

*[…]* I used to sleep at a bus stop alone *[…]* and so I did. Sometimes, when I slept on the street here in Fortaleza, I was hit by the police, because they caught me at night in an alley, because I lived in a slum, then they hit me *(E11)*. *[…]* Yes, I don’t sleep well on the street, those things of the night, of fear; so I often prefer to sleep during the day *(E13)*.

In this study, homeless adolescents sought a place considered safe and hidden, where they could spend the night. Taking care of oneself on the street involves the possibility of sleeping day and night, although there are no doors and windows on the streets for safety and privacy^14^.

Using a house to sleep is mentioned by some adolescents as challenging. Whenever they can, they rent a place to rest:

*[…]* Every day I come to fight, I have a daughter, I pay the rent, thank God every day I make “some money” and take it home *(E15)*. *[…]* No. I’m renting a small room so I can sleep *[...]*. I used to sleep on squares. There was no place to sleep, now I managed to rent a place *(E8)*.

Regarding the place where they sleep, who they sleep with, and such association with age group, gender identity, and race, no statistically significant association was observed between these variables (Table 4).

**Table 4:** Bivariate association analysis of the variables: where they sleep, who they sleep with, and sleep quality/quantity, rent, street food, and personal hygiene. Fortaleza, CE, Brazil, 2021 (n=19)

Characteristic	Test	Value	Degrees of freedom	P-value
Where they sleep/Age group	Pearson’s chi-square	1.377	1	0.241
	Likelihood ratio	1.336	1	0.248
	Linear-by-linear association	1.305	1	0.253
	Number of valid cases	19		
Who they sleep with/Age group	Pearson’s chi-square	0.224	1	0.636
	Likelihood ratio	0.223	1	0.637
	Linear-by-linear association	0.212	1	0.645
	Number of valid cases	19		
Daily food/Age group	Pearson’s chi-square	0.046	1	0.829
	Likelihood ratio	0.047	1	0.829
	Linear-by-linear association	0.044	1	0.834
	Number of valid cases	19		
Place of personal hygiene/Age group	Pearson’s chi-square	4.484	2	0.106
	Likelihood ratio	4.270	2	0.118
	Linear-by-linear association	3.196	1	0.074
	Number of valid cases	19		
Where they sleep/Gender identity	Pearson’s chi-square	3.292	2	0.193
	Likelihood ratio	4.722	2	0.094
	Linear-by-linear association	1.498	1	0.221
	Number of valid cases	19		
Who they sleep with/Gender identity	Pearson’s chi-square	5.276	2	0.072
	Likelihood ratio	6.439	2	0.040
	Linear-by-linear association	4.975	1	0.026
	Number of valid cases	19		
Daily food/Gender identity	Pearson’s chi-square	2.122	2	0.346
	Likelihood ratio	3.120	2	0.210
	Linear-by-linear association	0.834	1	0.361
	Number of valid cases	19		
Place of personal hygiene/Gender identity	Pearson’s chi-square	2.486	4	0.647
	Likelihood ratio	3.470	4	0.482
	Linear-by-linear association	0.707	1	0.400
	Number of valid cases	19		
Where they sleep/Race	Pearson’s chi-square	0.101	1	0.750
	Likelihood ratio	0.105	1	0.746
	Linear-by-linear association	0.096	1	0.756
	Number of valid cases	19		
Who they sleep with/Race	Pearson’s chi-square	0.130	1	0.719
	Likelihood ratio	0.128	1	0.720
	Linear-by-linear association	0.123	1	0.726
	Number of valid cases	19		
Daily food/Race	Pearson’s chi-square	0.377	1	0.539
	Likelihood ratio	0.368	1	0.544
	Linear-by-linear association	0.357	1	0.550
	Number of valid cases	19		
Place of personal hygiene/Race	Pearson’s chi-square	1.360	2	0.507
	Likelihood ratio	2.167	2	0.338
	Linear-by-linear association	1.010	1	0.315
	Number of valid cases	19		

Regarding food, 12 (63.2%) adolescents buy their food and 7 (36.8%) eat at an accessible restaurant, but their statements show they feel hungry, going through several experiences on the streets in order to find food:

[…] *When it’s early in the morning, he gives some food for me to eat. I buy coffee for myself. Then at lunch when I don’ pick some food here, then only at 3pm. Then at 3pm, I go there and pick it up, because it’s closing time, there’s always food leftover, not from the people who eat it, but there’s always that food left over and he gives it to homeless people. Then I live this way* (E16). [...] *So, I looked for food in the garbage and I ate food from the garbage, things I wouldn’t eat, I’m not going to humiliate myself, because I’ve humiliated, I was small, I’ve humiliated, because he was from the street. So, I’m going through this today, I know he suffered* (E19).

On the other hand, personal hygiene is one of the most difficult activities to be performed by adolescents who are homeless. Our findings show that 11 (57.9%) adolescents perform their personal hygiene in public toilets, such as containers. Then, hygiene is rarely performed by homeless people. With the absence of actions from the government, adolescents use informal resources, such as the street itself, but with more difficulties:

[…] *I can’t have a shower properly. It was kind of difficult* (E1). […] *I took a bath like this: there was a spout* […]*. There was a big rock, and up there, there was a restaurant and a big pipe of water that kept running down. The flow was strong, it fell hard, then I went there and got wet and as I had no clothes to change, I waited for them to dry, and I felt very cold* (E3). […] *There is a public toilet* [container]*, it offers free baths to people living on the streets* (E13).

According to the statements of adolescents, there are two bath units, called *Higiene Cidadã*, which are open every day from 9 am to 7 pm. One of the units is located on the beach and has 20 bathrooms with showers and space for personal hygiene. The other is a container in downtown, which offers 12 bathrooms, external sinks, and drinking fountains. These areas also have access to food and water. Each unit receives on average 100 people every day.

## Discussion

This study aimed to identify health inequalities of homeless adolescents based on the conceptual framework of social determinants of health. A very critical reality was observed among these adolescents, who were deprived of education, income, housing, food, and human dignity. They were aged 16 to 19 years, in agreement with a national study that analyzed the socioeconomic and demographic profile of adolescents who are homeless, from the perspective of sociocultural conditions^23^, with the presence of cisgender male and transgender male adolescents. These data also agree with another study conducted in downtown Toronto, Canada, in 2018, which reports an increase in the LGBTQIA+ population living on the streets^24^.

Regarding skin color, most adolescents reported brown and black skin. A social conjuncture linked with structural racism is observed, similar to that found in a Brazilian study, in which 80.9% of the participants were brown and black^21^ ^-^ ^23^.

When asked about how long adolescents had been homeless, they reported periods of more than six months, in agreement with estimates of the homeless population in Brazil. The number of people who are homeless has increased due to the COVID-19 pandemic crisis, as seen in Nova Scotia (Cape Breton), mainly due to economic issues, including increased unemployment and poverty^25^ ^-^ ^27^.

The contexts in which homeless adolescents are inserted and the way they live show different care practices. The health system and well-being are key factors for the development of society and to achieve equity in health. In this sense, the SDH approach helps understand the distribution of health resources and how care provision has promoted social justice and the access to services for the vulnerable population.

The fact that society has values of equality and social justice, as well as health services to fight against health inequities, are clear indicators that these aspects must be analyzed in scientific studies in order to produce evidence for public policies and internal coherence of these policies and their implementation in various sectors^28^ ^-^ ^30^.

Thus, understanding the concept of health inequalities is essential for health care. Self-care is strongly related to fulfilling health needs, which are seen not as the absence of illnesses^30^, but within the intercultural logic and intersubjectivity of human existence and dignity.

Inequalities express and are closely related to conditions of vulnerability; therefore, they must be seen from the subjectivity of the participants. Based on this perspective, homeless adolescents are heterogeneous, with their own characteristics, such as values, meanings, attributes, personal structure, survival strategies, and living conditions. These different characteristics involve various needs due to inequalities.

It is relevant to consider that poverty, among the several possible causes, does not allow individuals to meet their needs due to inequalities, as well as having a home to sleep, proper personal hygiene practices, food, and access to health. This context affects their physical, psychological, and social development^31^ ^-^ ^32^ and, consequently, can influence the continuity of adolescents on the streets, as reported in this study.

We can say the various vulnerabilities of adolescents became worse during the pandemic. Those who were permanently on the streets could not comply with the rule that was in force during the quarantine phase, which required everyone to “stay at home.” Staying at home with social distancing presupposes having a home, and our study found no option provided by the public power for this situation^21^.

In this perspective, sleeping on the streets - in places such as the beach, under overpasses, on squares, and at bus terminals - represents a complex reality that can affect the health of adolescents. Similar locations were identified in a longitudinal study conducted with street children and adolescents in three Brazilian capitals^33^.

Sleeping on the streets is a practice that generates doubts, distress, fears, and the search for adequate (safe) places by the participants. They reported a preference for sleeping during the day for safety reasons, in agreement with a study conducted with 251 homeless people (aged 20 to 60 years) in São Paulo, which showed that 65% claimed feeling unsafe on the streets and 45.7% said they had suffered aggression in the last month^34^. Another study with 244 homeless adults in Dallas, Texas, United States, showed that sleeping just a few hours is an aspect that influences the health/disease process of these people, resulting in mood disorders and a poor health self-assessment^35^.

Adolescents who are homeless are subject to a vulnerable and hostile environment, involving situations of violence, hunger, and fear at sleep time, due to SDH, lack of housing, money, ID documents - critical elements of citizenship- and obstacles to receiving health care.

As for eating on the streets, this study found participants have trouble maintaining healthy eating habits. Their access to food is poor and does not meet the daily energy and nutritional needs of an adolescent, compromising their health and well-being. Similar results were found in an ethnographic study conducted in Toronto, Canada, with 9 homeless adolescents (19-24 years), which highlighted the lack of food and obstacles to healthy eating habits^36^.

For the homeless population, food and personal hygiene are the main activities that require attention^37^. Obtaining food and a place for personal hygiene is even more challenging in the context of social distancing. The lack of access to these basic items leads to low self-esteem, situations of humiliation, illness, sadness, and violence.

Personal hygiene has never been considered so important among the economically privileged population, as a synonym for survival, as it has been today for the prevention of COVID-19. People are now required to wash their hands, take a shower, clean their homes and clothes. Care services for this population are few and, in Fortaleza, there is the *Higiene Cidadã* program, created due to the pandemic, have sufficient centers for all population.

Access to water, sanitary conditions, and hygiene are universal human rights, guaranteed by the Brazilian government in international agreements, which affect health and well-being. The UN 2030 Agenda determines that, by 2030, sanitary conditions (sewage treatment and toilets), drinking water and hygiene water (with soap supply) must be available to everyone^37^.

However, investments are required so this goal can be achieved. According to the World Health Organization, in Latin American countries, only 22% of the population has access to safe health services, while in North America and Europe, coverage reaches 78% of the population^38^. Whereas the challenge of achieving this goal is great for the general population, it is urgent for the homeless population.

Homeless adolescents face several obstacles in the attempt to fulfill their needs, especially regarding bath, which does not allow them to practice self-care, making them dirty, smelly, and victims of prejudice and distancing. Similar data were obtained in a study with homeless people, conducted in the cities of São Paulo and Salvador, in which the homeless condition associated with dirt, poor hygiene or bad smell is a factor that prevents the access to health services and increase social exclusion and prejudice^38^ ^-^ ^40^.

In this sense, the most prevalent diseases and health problems were malaria, mycosis, gastritis, respiratory problems, type 1 diabetes and mental illnesses, especially depression. Different results were obtained in a Japanese study and in a systematic review about homeless people, where the main diseases and health problems of this group included abuse of psychoactive substances, human immunodeficiency virus (HIV)/acquired immunodeficiency syndrome (AIDS) infection, mental disorders, and dental, dermatological and gastrointestinal problems^40^ ^-^ ^41^.

Our study also showed a limited number of health care programs and services for homeless adolescents; and when these services are available, the provision of care to the homeless population due to lack of an ID document is a commonly observed phenomenon. Due to the situation, the health care system must be structured to meet the needs of this population. The *Consultório na Rua* program was a milestone that generated progress to the provision of care to this social group. However, the program has only one office, which is not enough considering the high demand observed.

Our study highlights that the difficult access to health services as a result of the lack of ID documents is a reality experienced by adolescents who are homeless. The literature shows similar results from studies assessing the ID document requirement, restriction in meeting scheduled appointments of patients, limits in intersectoral practices, prejudice, among other aspects, creating poor bonds for people living on the streets^42^ ^-^ ^44^.

Some participants highlighted discrimination while seeking care in health services, such as the UPA. Studies indicate the delay and obstacles in seeking care is due to prejudice and discrimination related to individual’s condition of hygiene, clothing, smell, lack of ID document for identification and registration, in addition to previous episodes of poor service and even prohibition to enter public facilities^40^.

Based on the expanded concept of health, seen as a social phenomenon affected by the context where individuals and communities live, such as adolescents living on the streets^45^, covering all aspects of their individual and collective needs requires the adoption of a broader concept of care to enable the development of health strategies addressing problems and the intermediate determinants of health related to the health and well-being process.

Understanding the concept of inequalities is critical to health care work and nursing practice. Inequalities must be understood within the symbolic universe and intersubjectivity, considering they change according to the individual’s social position in the world, and the individual’s interpretation of the world and culture^45^ ^-^ ^46^; therefore, with a strategic understanding based on the subjectivity of individuals and social groups^47^.

In this perspective, it is important to consider future projects for nursing practice that is sensitive to the SDH of homeless adolescents, which cover the political, epistemological, and technical levels, inside and outside the health system. Nursing as a social practice proposes to understand and meet the needs of homeless adolescents, both individually and collectively, promoting their development and ensuring human dignity.

Our study had limitations, as data collection on the streets is a challenging process due to the mobility of adolescents and places of difficult access. Our study included convenience sampling, which does not allow the generalization of results.

Our study contributes to expanding the nursing and health knowledge and practice as it analyzed vulnerable populations, supporting the development of public policies for homeless adolescents, which guarantee, in the legal field, social rights, that is, the right to education, health, food, work, housing, leisure, security, social security, maternity and childhood protection. Our study also promotes reflections on new and more inclusive policies for adolescents, which allow them to be recognized as subjects of rights, requiring protection according to the law.

## Conclusion

Our study helps understand the context of homeless adolescents, their inequalities, and support the development of health strategies for equity and human dignity. Our findings show unique and challenging situations for adolescents, while promoting reflections for the provision of care to fulfill their health needs. In addition, our study attempted to highlight the inequalities of homeless adolescents aiming to build a health system with more equity, better performance of professionals, and treatment with more dignity for this population.
